# Economic and Clinical Burden of Herpes Zoster Among Patients With Inflammatory Bowel Disease in the United States

**DOI:** 10.1093/crocol/otad033

**Published:** 2023-07-13

**Authors:** David Singer, Philippe Thompson-Leduc, Deepshekhar Gupta, Sara Poston, Wendy Y Cheng, Siyu Ma, John E Pawlowski, Mei Sheng Duh, Francesca Devine, Azeem Banatwala, Emma Bernstein, Francis A Farraye

**Affiliations:** GSK, US Health Outcomes and Epidemiology—Vaccines, FMC Tower Suite 1700, 2929 Walnut Street, Philadelphia, PA 19104, USA; Analysis Group, Inc., Health Economics and Outcomes Research, 1190 Avenue des Canadiens-de-Montréal, Suite 1500, Montréal, QC H3B 0G7, Canada; Analysis Group, Inc., Health Economics and Outcomes Research, 1010 El Camino Real, Suite 310, Menlo Park, CA 94025, USA; GSK, US Health Outcomes and Epidemiology—Vaccines, FMC Tower Suite 1700, 2929 Walnut Street, Philadelphia, PA 19104, USA; Analysis Group, Inc., Health Economics and Outcomes Research, 111 Huntington Avenue, 14^th^ Floor, Boston, MA 02199, USA; GSK, US Health Outcomes and Epidemiology—Vaccines, FMC Tower Suite 1700, 2929 Walnut Street, Philadelphia, PA 19104, USA; Tufts Medical Center, 800 Washington Street, Boston, MA 02111, USA; GSK, Medical Affairs, FMC Tower Suite 1700, 2929 Walnut Street, Philadelphia, PA 19104, USA; Analysis Group, Inc., Health Economics and Outcomes Research, 111 Huntington Avenue, 14^th^ Floor, Boston, MA 02199, USA; Analysis Group, Inc., Health Economics and Outcomes Research, 151 West 42^nd^ Street, 23rd Floor, New York, NY 10036, USA; Analysis Group, Inc., Health Economics and Outcomes Research, 333 South Hope Street, 27^th^ Floor, Los Angeles, CA 90071, USA; Analysis Group, Inc., Health Economics and Outcomes Research, 111 Huntington Avenue, 14^th^ Floor, Boston, MA 02199, USA; Baylor University, Department of Political Science, One Bear Place #97276, Waco, TX 76798-7276, USA; Mayo Clinic, Division of Gastroenterology and Hepatology, 4500 San Pablo Road, Jacksonville, FL 32224, USA

**Keywords:** ulcerative colitis, Crohn’s disease, herpes zoster, healthcare resource utilization, costs

## Abstract

**Background:**

Patients with ulcerative colitis (UC) or Crohn’s disease (CD) are at increased risk of herpes zoster (HZ); however, relevant cost and healthcare resource utilization (HCRU) data are limited.

**Methods:**

We estimated HCRU (hospitalization, emergency department [ED], and outpatient visits) and costs in patients with UC or CD, with and without HZ, using administrative claims data (October 2015–February 2020). HCRU and costs (2020 US dollars) were compared at 1 month, 1 quarter, and 1 year after the index date, using propensity score adjustment and generalized linear models.

**Results:**

In total, 20 948 patients were included: UC+/HZ+ (*n* = 431), UC+/HZ– (*n* = 10 285), CD+/HZ+ (*n* = 435), and CD+/HZ– (*n* = 9797). Patients with HZ had higher all-cause HCRU rates and all-cause total healthcare costs relative to those without HZ. In the first month, adjusted incidence rate ratios (aIRRs) for hospitalizations and ED visits for patients with UC and HZ compared with UC alone were 2.87 (95% confidence interval [CI], 1.93–4.27) and 2.66 (95% CI,1.74–4.05), respectively; for those with CD and HZ, aIRRs were 3.34 (95% CI, 2.38–4.70) and 3.31 (95% CI, 2.32–4.71), respectively, compared with CD alone (all *P* < .001). Adjusted cost differences in UC and CD cohorts with HZ over the first month were $2189 and $3774, respectively, chiefly driven by higher inpatient costs. The incremental impact on HCRU and costs in cohorts with HZ predominantly occurred during the first quarter following diagnosis.

**Conclusions:**

HZ is associated with increased HCRU and costs in patients with UC and CD, especially shortly after diagnosis.

## Graphical Abstract

**Graphical Abstract:**
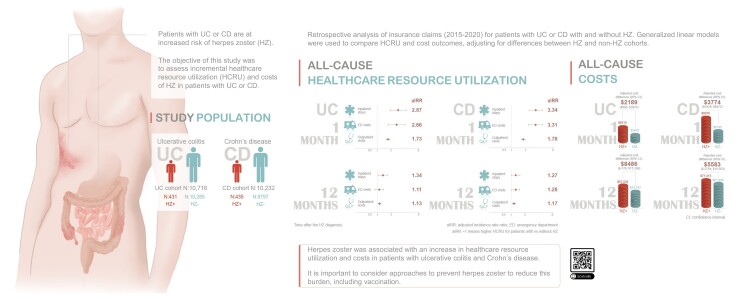

## Introduction

Herpes zoster (HZ) is relatively common, with over 1 million new cases each year in the United States (US).^1^ HZ risk increases with age,^2^ with a higher incidence in individuals aged ≥50 years. Recent US data from 2007 to 2018 indicate an incidence in individuals aged 51–60 years of 7.5 per 1000 person-years and 12.0 per 1000 person-years in those aged >70 years.^1^ HZ risk is higher in females and in patients who are immunocompromised.^2,3^ In patients who are immunocompromised, the risk of developing postherpetic neuralgia (PHN), an important complication affecting 5%–30% of patients, may also be greater.^4,5^ HZ represents a substantial economic burden, with estimated annual direct medical costs in individuals aged ≥50 years in the US (in 2013) of up to $1.9 billion (with substantial additional indirect costs).^6^

Patients with inflammatory bowel disease (IBD) are at higher risk of HZ, due to both the underlying disease and use of immunosuppressive therapies.^7–9^ In a recent US study, patients with IBD had a 1.7-times higher risk of HZ compared with those without IBD.^8^ There were no significant differences in risk between patients with ulcerative colitis (UC) or Crohn’s disease (CD), although other studies have reported a higher risk with CD.^7,10,11^ HZ risk is influenced by IBD therapy; receipt of thiopurines (alone or in combination with antitumor necrosis factor agents) and corticosteroids are each associated with greater risk of HZ than receiving only aminosalicylates (5-ASA) for disease control.^7,8,12^ Higher incidence of HZ is also observed in patients receiving Janus kinase (JAK) inhibitors.^13^

Given the increased risk of HZ in IBD patients, understanding the additional healthcare resource utilization (HCRU) and costs incurred in IBD patients with HZ is important. However, current data on HCRU and costs associated with HZ among IBD patients are limited. The aim of the present study was to estimate incremental (ie, differences in) HCRU and direct healthcare costs in UC and CD patients with HZ compared to those without HZ.

## Materials and Methods

### Study Design and Data Sources

This was a retrospective, longitudinal cohort study (GSK study identifier: VEO-000043) evaluating HCRU and associated costs in adults aged ≥18 years with UC or CD, with or without an HZ diagnosis. Data were sourced from the Optum’s deidentified Clinformatics Data Mart Database, which includes longitudinal, deidentified patient-level medical and pharmacy claims data from Medicare Advantage and commercial health plans. The study utilized data spanning the period from October 1, 2015, until February 28, 2020. Patients were assigned to 4 mutually exclusive cohorts on the basis of any prior HZ diagnosis: UC+/HZ+, UC+/HZ–, CD+/HZ+, and CD+/HZ–.

**Figure 1.**
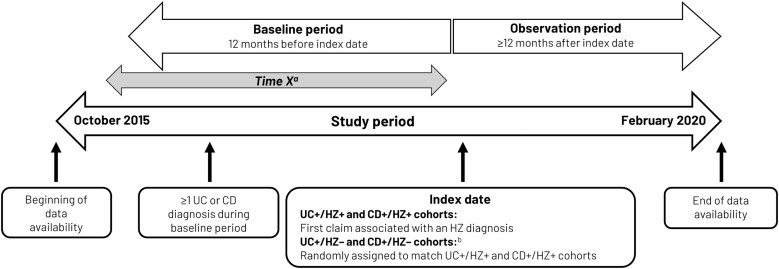
Study design. The study utilized data spanning the period from October 1, 2015 until February 28, 2020. The baseline period was defined as a 12-month period with continuous health-plan enrollment prior to the index date (between October 1, 2015, and February 28, 2018). The index date was defined as the date of the first HZ diagnosis (between October 1, 2016, and February 28, 2019), with the observation follow-up period spanning the 12-month period from the index date (ie, between October 1, 2017 and February 28, 2020). ^a^Time X was determined only for the UC+/HZ– and CD+/HZ– cohorts and represents a randomly selected value such that the distribution of X’s follow that of the pre-index eligibility in the respective UC+/HZ+ and CD+/HZ+ cohorts. ^b^The index date for the UC+/HZ– and CD+/HZ– cohorts was randomly assigned based on the time distribution from the start of continuous enrollment to index dates in their respective UC+/HZ+ and CD+/HZ+ cohorts. Abbreviations: CD, Crohn’s disease; HZ, herpes zoster; UC, ulcerative colitis. UC+/HZ+ and UC+/HZ–: patients with UC with and without HZ respectively; CD+/HZ+ and CD+/HZ–: patients with CD with and without HZ, respectively.

The study measured HCRU and costs over a 12-month period after an index date, which, for UC and CD patients with HZ, was defined as the date of the first HZ diagnosis (see below). For UC and CD patients without HZ, an index date was randomly assigned to match the distribution of time from the start of continuous enrollment to index dates of their respective UC+/HZ+ and CD+/HZ+ cohorts. The study baseline period was defined as a 12-month period with continuous health-plan enrollment immediately prior to the index date, with the observation period defined as the 12-month period following the index date with continuous health-plan enrollment (Figure 1).

### Study Population

**Figure 2.**
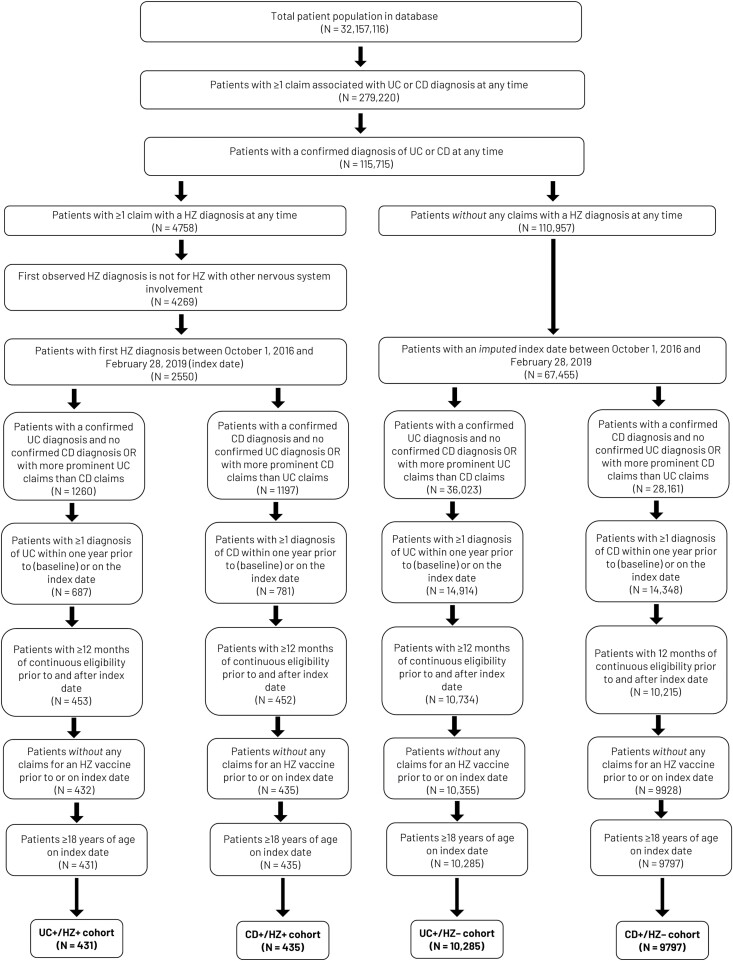
Study population and assignment in study cohorts. Abbreviations: CD, Crohn’s disease; HZ, herpes zoster; UC, ulcerative colitis. UC+/HZ+ and UC+/HZ–: patients with UC with and without HZ respectively; CD+/HZ+ and CD+/HZ–: patients with CD with and without HZ respectively.

Patients were required to be aged ≥18 years at the index date, grouped into mutually exclusive UC and CD cohorts, and identified on the basis of International Classification of Diseases, 10th Revision, Clinical Modification (ICD-10-CM) diagnosis codes (Table S1). Patients were required to have ≥2 claims on separate days with a diagnosis of UC (ICD-10-CM, K51) or CD (ICD-10-CM, K50), or at least 1 UC or CD diagnosis claim and at least 1 pharmacy claim for IBD-related medication (eg, 5-ASA, immunomodulators, biologics such as anti-TNF agents, or tofacitinib) within 30 days following the UC or CD diagnosis claim (see Figure 2 and Supplementary Appendix). For individuals with claims for both UC and CD, a majority-based algorithm of claims in the 12-month baseline period was used to allocate patients into the UC or CD cohorts^14,15^ (see Supplementary Appendix). The final assignment was based on claims associated with an HZ diagnosis. For inclusion in the respective UC+/HZ+ and CD+/HZ+ cohorts, patients were required to have had ≥1 claim with an HZ diagnosis (ICD-10-CM code B02; excluding HZ associated with other nervous system involvement [ICD-10 CM: B02.2]). Additionally, patients must have had no claims for any HZ vaccine prior to the index date. For UC+/HZ– and CD+/HZ– cohort inclusion, patients were required to have had no HZ disease between the beginning and end of continuous health-plan enrollment and no claims for any HZ vaccine prior to the index date.

### Covariates and Outcome Measures

Patient demographic characteristics were measured at index date; clinical characteristics were assessed during the baseline period. These included clinical management of UC and CD (ie, IBD-specific medication use, surgeries, or procedures; and markers of IBD severity), comorbidities, immunosuppressive medication use, and conditions associated with a potentially increased risk of HZ (see Supplementary Appendix). Overall comorbidity burden was measured using the modified Charlson–Quan comorbidity index (CCI).^16^

HCRU outcomes included hospital inpatient stays, emergency department (ED) visits, and outpatient (ie, hospital outpatient and physician office) visits. Costs included total costs, medical costs (all costs incurred on the medical benefit), and pharmacy costs (which included all costs incurred on the pharmacy benefit). Medical costs were further categorized according to the type of encounter, that is, inpatient, ED, or outpatient and other costs, including skilled nursing facilities, home care services, hospice, vision care, and provision of durable medical equipment.

All-cause HCRU and costs were calculated for each individual over the 12-month baseline period and across specific intervals during the observation period: 1 month, 1 quarter, and 1-year post-index. Costs were adjusted to 2020 US$ values using the medical care component of the US Consumer Price Index.^17^

### Statistical Analyses

Descriptive analyses were performed for all study variables. For continuous variables, means, standard deviations (SDs), and medians were calculated; frequency counts and percentages were calculated for categorical variables. Baseline characteristics were compared using standardized differences (see Supplementary Appendix), where standardized differences of 20%, 50%, and 80% suggest, respectively, small, medium, and large differences between cohorts.^18^

For HCRU comparisons between cohorts, the proportions of patients who used each type of medical service and the mean number of HCRU encounters/visits were reported. Corresponding incidence rates were calculated by dividing the number of encounters over the observation period by the patient-time observed and were reported per person per year (PPPY). For shorter time horizons, the average number of HCRU events per patient per month or per quarter was calculated. Propensity scores were calculated using logistic regressions with HZ status as the outcome and key baseline characteristics as predictors. Key baseline characteristics included were year of index date, age, sex, geographic region, insurance plan type, comorbidities/comorbidity index, immunosuppressive conditions, markers of IBD severity (eg, weight loss, malnutrition, anemia), surgeries and procedures, all-cause costs during the baseline period, and IBD-related medication. Adjusted incidence rate ratios (aIRRs) and 95% confidence intervals (CIs) were estimated using generalized linear models with a negative binomial distribution (to accommodate overdispersion) and log link, with propensity score and key baseline characteristics (ie, year of index date, age, sex, geographic region, insurance plan type, comorbidities/comorbidity index, immunosuppressive conditions, markers of IBD severity, surgeries and procedures, all-cause costs during the baseline period, and IBD-related medication) included in the models as covariates to account for potential baseline cohort differences. The variables included in propensity scores and additionally included as covariates in outcome models were the same. The use of covariate adjustment with propensity score as an additional covariate has been shown to provide reliable effect estimates across different datasets with more consistent performance compared with other propensity score methods such as stratification or inverse weight probability.^19^*P*-values were calculated using the negative binomial distribution (see Supplementary Appendix).

Mean costs (with SD) during the first 12 months of the observation period were reported on a PPPY basis and stratified by service use type. Costs incurred during the first month and cumulative costs over the first 3 months were also reported. Costs for each cohort were estimated using a 2-part model,^20^ incorporating doubly robust adjustment using propensity scores and key baseline characteristics within logistic regression and generalized linear models with a log-link and gamma distribution. Each model was fitted separately and their predictions combined to derive mean estimated costs. A recycled predictions approach was then used to estimate absolute adjusted cost differences between groups with and without HZ (see Supplementary Appendix). For adjusted cost differences, 95% CIs were estimated using nonparametric bootstrap procedures with 499 replications. All statistical analyses were conducted using the statistical software SAS 9.4 or SAS Enterprise Guide 7.1 (SAS Institute Inc., Cary, NC).

### Ethical Considerations

The study employed a retrospective cohort study design using a large administrative claims database. Data were deidentified and complied with the requirements of the Health Insurance Portability and Accountability Act. Institutional Review Board review and approval was therefore not required, as per United States Department of Health and Human Services regulation for the protection of human subjects in research (45 CFR 46, https://www.hhs.gov/ohrp/regulations-and-policy/regulations/45-cfr-46). The study has been conducted in accordance with the guiding principles of the Declaration of Helsinki. As only existing deidentified data have been analyzed and as patients have not been contacted during the course of this study, informed consent process is not applicable.

## Results

### Study Population and Baseline Characteristics

The study population was as follows: UC+/HZ+ (*n* = 431), UC+/HZ– (*n* = 10 285), CD+/HZ+ (*n* = 435), and CD+/HZ– (*n* = 9797). Full details of patient identification and assignment are shown in Figure 2. Selected baseline demographics, clinical characteristics, HCRU, and costs in the year prior to the index date are shown in Table 1 (see also Tables S2 and S3). In each cohort, the proportion of female patients was higher (54.5%–62.1%). Patients with HZ were relatively older than those without HZ (Table S2) and had a slightly greater overall comorbidity burden (as assessed by CCI scores; Table 1). The prevalence of specific comorbidities varied among cohorts, although standardized differences were small (<20%; Table S2). Use of IBD-related medications in the 12-month pre-index period ranged from 55.6% to 59.1% across cohorts. A relatively higher proportion of patients with HZ received systemic steroids during this period than those without HZ; a similar pattern was observed for thiopurine use. Some small differences in HCRU and healthcare costs were observed between the UC+/HZ+ and UC+/HZ– cohorts and between the CD+/HZ+ and CD+/ HZ– cohorts, although standardized differences were <20% (Tables 1 and S3).

**Table 1.** Baseline demographics and clinical characteristics and prior HCRU use and costs.

	UC			CD		
	UC+/HZ+ (*N* = 431)	UC+/HZ– (*N* = 10 285)	Standardized difference^a^	CD+/HZ+ (N = 435)	CD+/HZ– (*N* = 9797)	Standardized difference^a^
Demographics						
Age at index date, years, mean ± SD	65.3 ± 15.1	60.0 ± 17.5	32.2%	61.0 ± 17.0	56.0 ± 18.0	28.2%
18–29, *n* (%)	6 (1.4)	644 (6.3)	25.4%	28 (6.4)	1034 (10.6)	14.8%
30–39, *n* (%)	32 (7.4)	1145 (11.1)	12.8%	38 (8.7)	1190 (12.1)	11.2%
40–49, *n* (%)	40 (9.3)	1251 (12.2)	9.3%	49 (11.3)	1348 (13.8)	7.5%
18–49, *n* (%)	78 (18.1)	3040 (29.6)	26.9%	115 (26.4)	3572 (36.5)	21.6%
50–64, *n* (%)	104 (24.1)	2200 (21.4)	6.5%	105 (24.1)	2480 (25.3)	2.7%
≥65, *n* (%)	249 (57.8)	5045 (49.1)	17.5%	215 (49.4)	3745 (38.2)	22.6%
Female, *n* (%)	258 (59.9)	5602 (54.5)	10.9%	270 (62.1)	5518 (56.3)	11.7%
Geographic region, *n* (%)						
South	141 (32.7)	4275 (41.6)	18.3%	176 (40.5)	4159 (42.5)	4.0%
West	111 (25.8)	2134 (20.7)	11.8%	99 (22.8)	1733 (17.7)	12.6%
Midwest	124 (28.8)	2359 (22.9)	13.3%	110 (25.3)	2591 (26.4)	2.6%
Northeast	55 (12.8)	1501 (14.6)	5.3%	50 (11.5)	1305 (13.3)	5.5%
Unknown	0 (0.0)	16 (0.2)	5.6%	0 (0.0)	9 (0.1)	4.3%
Insurance type, *n* (%)						
Medicare advantage	253 (58.7)	5354 (52.1)	13.4%	251 (57.7)	4729 (48.3)	18.9%
Commercial	178 (41.3)	4931 (47.9)	13.4%	184 (42.3)	5068 (51.7)	18.9%
Clinical characteristics						
CCI, mean ± SD	1.6 ± 2.2	1.1 ± 1.7	25.2%	1.4 ± 1.8	1.2 ± 1.7	14.4%
Use of IBD-related medications, *n* (%)	246 (57.1)	5719 (55.6)	3.0%	257 (59.1)	5705 (58.2)	1.7%
5-ASA	144 (33.4)	3981 (38.7)	11.0%	67 (15.4)	1745 (17.8)	6.5%
Systemic steroids	72 (16.7)	1284 (12.5)	12.0%	86 (19.8)	1471 (15.0)	12.5%
Tumor necrosis factor inhibitors	55 (12.8)	1122 (10.9)	5.7%	103 (23.7)	2490 (25.4)	4.0%
Thiopurines	48 (11.1)	790 (7.7)	11.8%	75 (17.2)	1151 (11.7)	15.6%
Total healthcare costs ($) PPPY, mean ± SD	$58 230 ± 83 680	$49 526 ± 84,736	10.3%	$72 849 ± 87 582	$65 983 ± 87 112	7.9%

^a^Standardized differences of 20%, 50%, and 80% suggest small, medium, and large differences between cohorts, respectively.^18^

Abbreviations: 5-ASA, aminosalicylate; CCI, modified Charlson–Quan comorbidity index; CD, Crohn’s disease; ED, emergency department; HCRU, healthcare resource utilization; HZ, herpes zoster; IBD, inflammatory bowel disease; PPPY, per person per year; SD, standard deviation; UC, ulcerative colitis.

UC+/HZ+ and UC+/HZ–: Patients with UC with and without HZ, respectively; CD+/HZ+ and CD+/HZ–: patients with CD with and without HZ, respectively.

### Healthcare Resource Utilization

**Figure 3.**
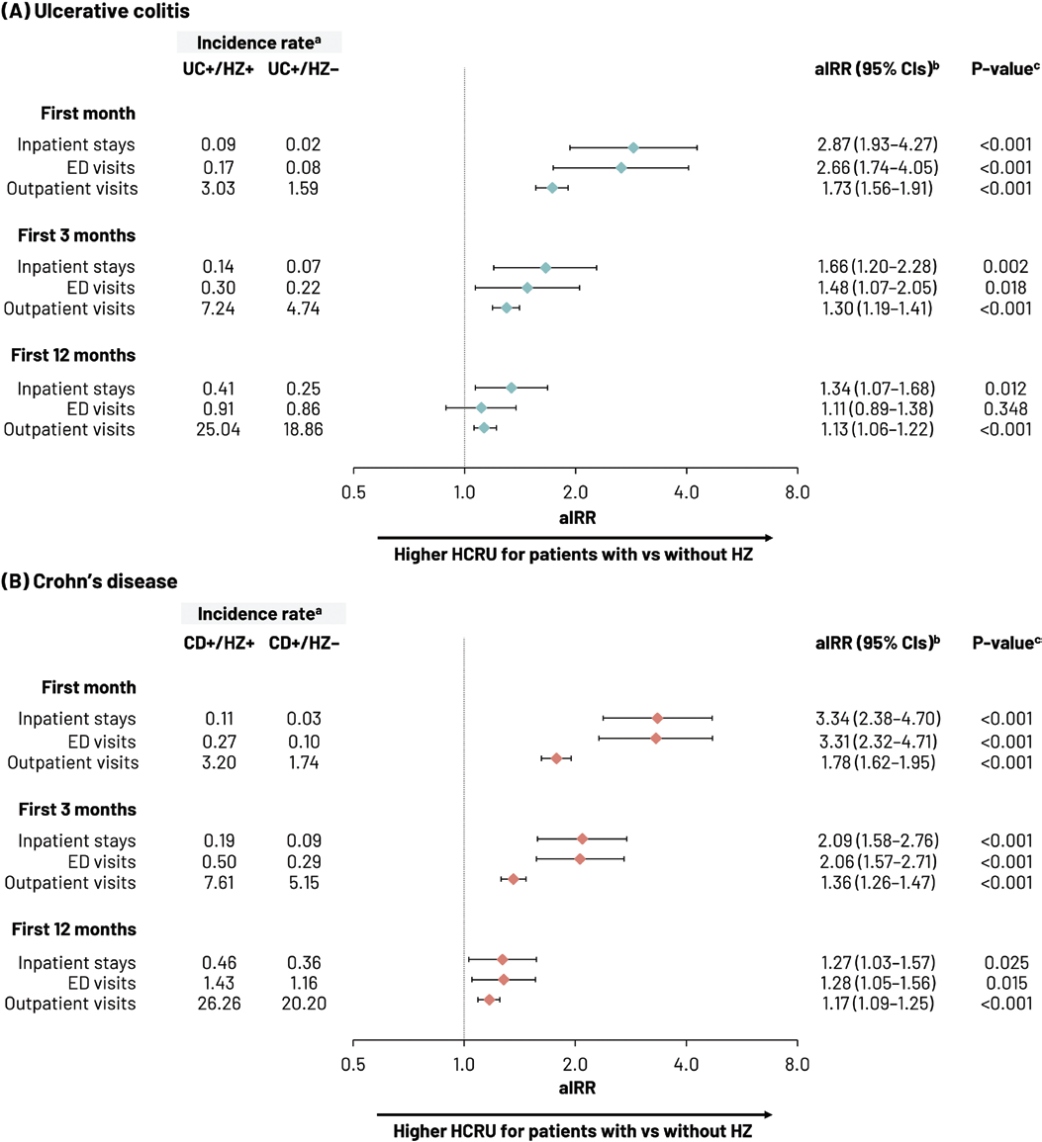
Incidence rates and adjusted incidence rate ratios for all-cause medical service use for UC (A) and CD patients (B) with and without HZ during the first year of observation. ^a^Incidence rates for HCRU (inpatient stays, ED, and outpatient visits) were calculated by dividing the number of encounters over the observation period by the patient-time observed, and reported on a PPPY basis for the first year of the observation period. For the first month and first quarter time horizons, the mean number of HCRU events per patient were described. ^b^aIRRs were calculated using generalized linear models assuming a negative binomial distribution and log link, accounting for the propensity score of being in the UC+/HZ+ or CD+/HZ+ cohorts and relevant baseline characteristics. ^c^*P*-values were calculated using the negative binomial distribution. Abbreviations: aIRRs, adjusted incidence rate ratios; CD, Crohn’s disease; CI, confidence interval; ED, emergency department; HCRU, healthcare resource utilization; HZ, herpes zoster; PPPY, per person per year; UC, ulcerative colitis. UC+/HZ+ and UC+/HZ–: patients with UC with and without HZ respectively; CD+/HZ+ and CD+/HZ–: patients with CD with and without HZ respectively.

During the 12-month post-index date period, UC and CD patients with HZ had a greater number of HCRU events than those without HZ, especially during the first month (Figure 3 and Table S4). At 1 month, aIRRs for hospitalizations, ED visits, and outpatient visits were 2.87 (95% CI, 1.93–4.27), 2.66 (95% CI, 1.74–4.05), and 1.73 (95% CI, 1.56–1.91), respectively, for the UC+/HZ+ cohort versus the UC+/HZ– cohort (all *P* < .001). For the CD+/HZ+ cohort, aIRRs for hospitalizations, ED visits, and outpatient visits were 3.34 (95% CI, 2.38–4.70), 3.31 (95% CI, 2.32–4.71), and 1.78 (95% CI,1.62–1.95), respectively, compared with the CD+/HZ– cohort (all *P* < .001). This greater HCRU by patients with HZ relative to those without HZ continued throughout the study follow-up periods (Figure 3 and Table S4). aIRRs for hospitalizations remained higher in those patients with CD and UC with HZ across the entire 12 months. Similar patterns were observed in patients with UC and CD with HZ for additional visit categories, including ED visits, although at some time points for some categories, these higher adjusted rates were not statistically significant (Figure 3).

### Healthcare Costs

**Figure 4.**
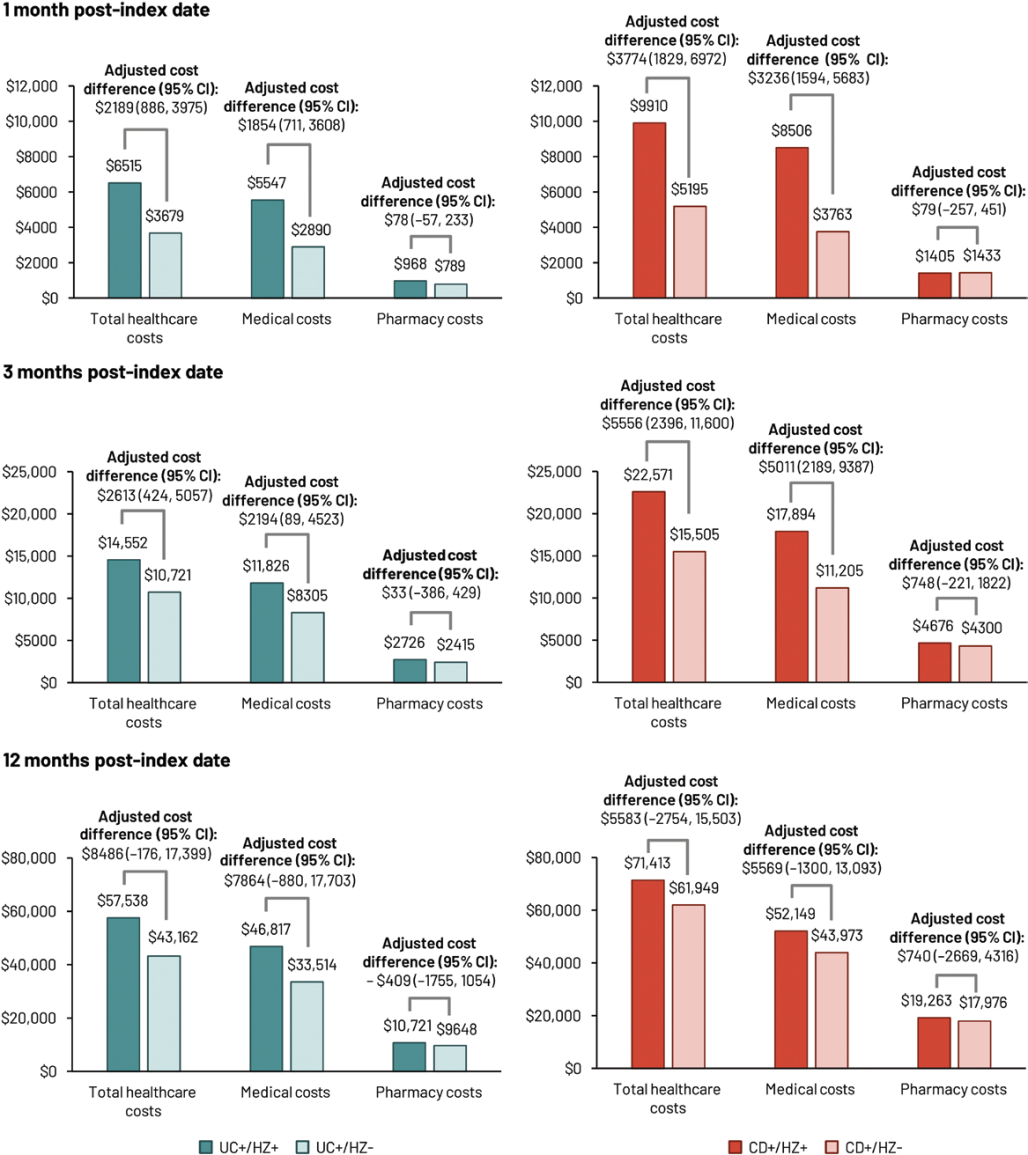
All-cause healthcare costs in UC and CD patients with and without HZ. Total costs represent all medical costs plus pharmacy costs. Medical costs represent total HCRU costs (inpatient stays and ED, outpatient, and other visits). Costs and adjusted cost differences were estimated using a two-part modeling approach, using logistic regression and a generalized linear model with a recycled predictions approach; 95% CIs were estimated from nonparametric bootstrap procedures with 499 replications. Abbreviations: CD, Crohn’s disease; CI, confidence interval; ED, emergency department; HCRU, healthcare resource utilization; HZ, herpes zoster; UC, ulcerative colitis. UC+/HZ+ and UC+/HZ–: patients with UC with and without HZ, respectively; CD+/HZ+ and CD+/HZ–: patients with CD with and without HZ, respectively.

Patients with UC and CD who developed HZ incurred greater all-cause total healthcare costs relative to those without HZ (Figure 4 and Table S5). At 1 month, mean all-cause total healthcare costs in UC patients with versus without HZ were $6515 versus $3679 (adjusted cost difference = $2189 [95% CI, 886, 3975]). For CD patients with versus without HZ, at 1 month, mean all-cause total healthcare costs were $9910 versus $5195 (adjusted cost difference = $3774 [95% CI, 1829, 6972]). Mean all-cause medical costs with versus without HZ were also higher at 1 month (**Figure 4**). These medical and total cost differences in the first month after index were driven by higher inpatient costs in the HZ cohorts (Table S5).

Cumulative all-cause total healthcare costs and medical costs in patients with HZ were also significantly higher at 3-month post-index date (Figure 4). After 12 months, cumulative mean all-cause total healthcare and medical service PPPY costs remained greater in UC and CD patients with HZ, although cost differences over the entire 12 months were not statistically significant (Figure 4 and Table S5). For UC, mean all-cause total healthcare costs in patients with versus without HZ over the 12-month observation period were $57 538 versus $43 162 (adjusted cost difference = $8486 [95% CI, –176, 17 399]). For CD, mean all-cause total healthcare costs in patients with versus without HZ during the same period were $71 413 versus $61 949 (adjusted cost difference = $5583 [95% CI, –2754, 15 503]). All-cause medical costs in patients with versus without HZ were also higher. Inpatient PPPY costs for CD patients with HZ were significantly higher after 12 months compared with those without HZ: $25 327 versus $18 360 (adjusted cost difference = $5826 [95% CI, 262, 12 048]).

There were no substantial differences in pharmacy costs between cohorts at any time during the 12-month observation period.

## Discussion

We found that patients with UC and CD who developed HZ generally had higher rates of HCRU and higher healthcare costs than patients without HZ. HCRU rates and cost differences were greatest within the first month following HZ diagnosis, with adjusted differences in total healthcare costs in UC and CD patients with HZ being $2189 and $3774, respectively, relative to those without HZ. Cost differences during the first month were driven by increased medical costs (chiefly greater inpatient costs); there were minimal differences in pharmacy costs; not entirely unexpected as many HZ-related medications (eg, antivirals, analgesics) are available as relatively low-cost generics. While aIRRs for HCRU showed significant relative differences in the use of healthcare services, absolute incidence rates and incidence rate differences were small for inpatient and ED encounters, which were somewhat rare in this population. Despite these small absolute differences, HZ might still have a large impact on HCRU and costs given the size of the IBD population.

HCRU differences remained significant after 3 months and costs remained higher. While absolute costs remained greater in UC and CD patients with HZ after 12 months, adjusted cost differences were not statistically significant with 95% CIs around these point estimates crossing zero. This is consistent with previous studies reporting greater costs in the initial period following the development of HZ and subsequent attenuation of cost differences.^21–24^ While some patients may experience longer-lasting complications (eg, PHN) which may contribute to longer-term cost increases, the cost impact can be difficult to measure in an overall sample of patients with HZ.^23,24^ Throughout our study, observed mean costs were high, with a high degree of variability. This, together with the relatively small numbers of patients with HZ (and possible underpowering), may have impacted our ability to detect meaningful cost differences over 12 months.

In the present study, mean ± SDs PPPY all-cause healthcare costs during the 12-month observation period were $43 162 ± 73 558 and $61 949 ± 90 701, respectively, for those UC and CD patients without HZ. Previous studies have reported lower average costs for UC and CD patients ($18 000–$25 000).^14,25,26^ This may in part reflect differences among study populations. Previous studies specifically included incident IBD cases while the current study did not, meaning many patients in our study may have had prevalent and potentially more severe disease. Our study also included a substantial proportion of older patients, with more than 40% of patients aged ≥65 years, and comorbidity burden was higher than in previous studies.^14,25,26^ The resulting study population may be more relevant for the research objective, that is, evaluation of a potential impact of HZ on HCRU and costs. Indeed, older individuals and individuals with comorbidities are at higher risk of HZ, and therefore, HZ is likely to be more frequent in the present study population compared with a population of incident IBD patients.

Minor differences in HCRU were apparent in the UC and CD cohorts. HZ was often associated with a numerically greater increase in HCRU in patients with CD than in those patients with UC in this study (as reflected in slightly higher aIRRs for HCRU use in the CD cohorts than estimated for the UC cohorts). With some evidence that patients with CD have a slightly greater risk of HZ than those with UC,^7,10,11^ it is possible that HZ severity is also greater in patients with CD compared with patients with UC, resulting in greater HCRU. While our study was not designed to address this research question, and any interpretations on this topic should be made with caution in the context of this study, it may represent an important future research direction.

To the best of our knowledge, no previous studies have examined the incremental cost in IBD patients with versus without HZ, although several studies have evaluated HZ-associated costs in various US patient populations (immunocompetent, immunocompromised, and with specific comorbidities, for example, chronic obstructive pulmonary disease [COPD] and type 2 diabetes).^21–24,27,28^ In a matched cohort study of immunocompetent patients aged ≥50 years (with and without HZ), adjusted incremental mean total all-cause healthcare costs (2013 US$) in HZ patients were $1809 in the first 12 months, with the majority of costs ($979) incurred during the acute phase of HZ (ie, in the first month).^22^ A similar study in immunocompromised patients (chiefly patients with human immunodeficiency virus, cancer, and those receiving immunosuppressive therapy) reported incremental healthcare costs of HZ observed during the first quarter in immunocompromised populations that were generally similar to those observed in this study, ranging from $2549 to $4297, with the exception of bone marrow/stem cell transplant patients, a particularly immunocompromised group, having an estimated incremental cost of HZ of $13 332.^21^ Among patients with COPD, higher all-cause healthcare costs in patients with HZ over 12 months have been reported.^27^ Similar to the present study, the observed cost differences in these populations were principally incurred during the period shortly after HZ diagnosis.^21–24,27^

Our study has several limitations. We did not specifically identify HZ-related HCRU and costs in our HZ+ cohorts but made adjusted comparisons of all-cause costs and estimated adjusted incremental costs associated with HZ in IBD patients. While this approach does not directly measure HZ-specific costs, it may account for costs that were associated with sequelae of HZ but not coded as related to HZ in claims. Indirect costs were not included in our analysis, so our findings likely underestimate the total incremental costs associated with HZ in patients with UC or CD. The impact of HZ on health-related quality of life was not considered in this study. Given the substantial burden of HZ in IBD patients, cost-effectiveness studies may provide complementary evidence on the value of HZ vaccination in this population.

This study was conducted using administrative claims data and has limitations common to all such retrospective claims database analyses, such as billing record miscoding, missing data, and lack of detailed clinical data. While prior HZ vaccination was an exclusion criterion, this was ascertained based upon vaccine codes identified prior to the index date. Although it remains possible that some had received HZ vaccination at some point prior to the patient’s period of continuous enrollment available for this study, there was relatively low HZ vaccine coverage in the general population during the study period, with data from the Centers for Disease Control and Prevention indicating that HZ vaccination coverage in all adults aged ≥50 years in the US was approximately 26% in 2019.^29^ As such, in all likelihood, relatively few patients with prior HZ immunization would have been included between the low coverage and the study exclusion criteria.

Additionally, there is some evidence that HZ-related HCRU and costs can be incurred in the HZ prodromal phase,^30^ which may have occurred prior to the HZ index date in some cases. Consequently, costs in our HZ cohorts may be underestimated, as some may have been incurred before the index date. Despite the doubly robust analytical approach that separately included baseline characteristics and propensity scores in outcome models, residual confounding may have influenced our findings. While some methods, such as high-dimensional propensity score matching, aim at reducing residual confounding due to unknown or unobserved variables by incorporating a large number of observed variables into the propensity score and matching on this, matching was deemed not to be appropriate for our data set.^31^ Indeed, propensity score matching would have eliminated a large number of data points given the large number of individuals without HZ, thereby losing valuable information and reducing the generalizability of our results by matching, even if a ratio of 1:3–5 was feasible.^19^

Nevertheless, as most studies on HZ in IBD have focused on HZ risk, our findings provide additional useful context. The higher HCRU and costs observed in the 3 months following HZ diagnosis, and the generally greater HCRU and costs apparent throughout the 12-month observation period, provide support for the use of HZ vaccination as part of a broader preventive health approach for IBD.^32–35^ An effective recombinant zoster vaccine (RZV) is available and is recommended by the Advisory Committee on Immunization Practices (ACIP) for all immunocompetent individuals over the age of 50 years.^36^ ACIP recently recommended the use of RZV in individuals aged ≥19 years who are or will be immunodeficient or immunosuppressed because of disease or therapy.^37^ Supportive data for the benefit of RZV in IBD patients are emerging, with recent studies reporting that 2-dose RZV immunization was associated with a significantly lower risk of HZ in IBD patients aged ≥50 years.^38,39^ Safety data indicate that local and systemic adverse reactions in IBD patients after RZV vaccination are comparable with those seen in immunocompetent individuals, with a low rate of IBD flares.^40^

In conclusion, our study shows that HZ is associated with significant incremental HCRU and costs in patients with IBD during the time following a diagnosis. Patients may benefit from vaccination to reduce disease burden and costs.

## Supplementary Material

otad033_suppl_Supplementary_Material

## Data Availability

GSK makes available anonymized individual participant data and associated documents from interventional clinical studies which evaluate medicines, upon approval of proposals submitted to www.clinicalstudydatarequest.com. To access data for other types of GSK sponsored research, for study documents without patient-level data and for clinical studies not listed, please submit an enquiry via the website.
