# Supplementary material for: Economic and Clinical Burden of Herpes Zoster Among Patients With Inflammatory Bowel Disease in the United States
Source: Crohns Colitis 360. 2023 Jul 13;5(3):otad033. doi: 10.1093/crocol/otad033 (PMC10368335; doi:10.1093/crocol/otad033)
Supplement: otad033_suppl_Supplementary_Material [file otad033_suppl_supplementary_material.pdf]

Supplementary Material

Economic and Clinical Burden of Herpes Zoster among Patients with Inflammatory Bowel Disease in the United States

Supplementary Information

Codes Used to Identify Patients and Medication Use in Study Cohorts

Clinical outcomes of interest were identified using relevant diagnostic and medication codes. Clinical diagnoses were identified using International Classification of Diseases-10 Clinical Modification (ICD-10-CM) codes. Pharmacy claims and medication use are identifiable via relevant medication codes: Current Procedural Terminology (CPT); Healthcare Common Procedure Coding System (HCPCS); Generic Product Identifier (GPI); and National Drug Code (NDC); procedural codes from the International Classification of Diseases, 10th Revision, Procedure Classification System (ICD-10-PCS) are also available.

Codes used to identify anonymized individuals with clinical diagnoses of interest, i.e., with ulcerative colitis (UC), Crohn’s disease (CD), and herpes zoster (HZ), for selection and inclusion in the study cohorts are shown in Supplementary Table 1. A more complete list of codes used to identify pharmacy claims is shown in Supplementary Table 6, at the end of this Supplementary Appendix.

Eligible individuals were patients ≥18 years of age at the index date, with a diagnosis of UC or CD identified using relevant ICD-10 CM codes. Based on previously validated claims algorithms patients with UC (K51) or CD (K50) were grouped into two mutually exclusive patient cohorts (UC and CD). For individuals with claims for both UC and CD, a majority-based algorithm of claims in the 12-month baseline period was used to allocate patients into the UC or CD cohorts, as used in previous studies.[1, 2] Patients were classified as having CD if (a) the number of CD-related inpatient (IP) admissions was higher than the number of UC-related IP admissions; (b) there was an equal number of CD- and UC-related IP admissions but more CD-related outpatient (OP) visits than UC-related OP visits; or (c) there was an equal number of CD- and UC-related IP admissions and OP visits but the most recent claim prior to the HZ index date was for CD.

Table S1. Codes used to identify patients in study cohorts

Code Type	Code	Condition
ICD-10-CM	K51	UC
ICD-10-CM	K50	CD
ICD-10-CM	B02.2	HZ with other nervous system involvement
ICD-10-CM	B02.3	HZ ocular disease
ICD-10-CM	B02.7	Disseminated HZ
ICD-10-CM	B02.8	HZ with other complications
ICD-10-CM	B02.9	HZ without complications
ICD-10-CM	B02.0	HZ encephalitis
ICD-10-CM	B02.1	HZ meningitis

CD, Crohn’s disease; HZ, herpes zoster; ICD-10-CM, International Classification of Diseases, 10th Revision, Clinical Modification; UC, ulcerative colitis

Data Analyses

Comparison of Baseline Characteristics

Standardized differences were used to compare baseline characteristics between cohorts. For continuous variables, the standardized difference was calculated by dividing the absolute difference in means of each cohort comparison by the pooled standard deviation of both groups (where the pooled standard deviation was the square root of the average of the squared standard deviations). For categorical variables with two levels, the standardized difference was calculated using the following equation: $abs(P1-P2)/\sqrt{p(1-p)})$, where $p = (P1+P2)/2$; P1 was the respective proportion of participants in the UC+/HZ+ and CD+/HZ+ cohorts; and P2 was the respective proportion of participants in the UC+/HZ– and CD+/HZ– cohorts. Standardized differences of 20%, 50%, and 80% suggest small, medium, and large differences between cohorts, respectively.[3]

Incidence Rate Ratio and Propensity Score Adjustment

Adjusted incidence rate ratios (aIRRs) and 95% confidence intervals (CIs) were estimated using generalized linear models (assuming a negative binomial distribution and log link, adjusting for potential confounders using the patients' propensity score as a covariate in addition to other baseline variables to adjust for baseline differences between cohorts). *P*-values were calculated using the negative binomial distribution.

In this approach, logistic regression was used to estimate a patient's propensity (or probability) of being in their cohort, based on their demographics on the index date (i.e., age, gender, geographic region, and insurance type), clinical characteristics (including baseline comorbidities and modified Charlson–Quan comorbidity index scores, baseline inflammatory bowel disease medication use, and prior clinical procedures) and healthcare costs incurred in the 12 months prior to the index date.

Cost Estimations and Adjusted Cost Differences

Healthcare costs for each of the study cohorts were estimated using a two-part model, an established method used in health economics to accommodate any skewed distribution for positive costs and any significant proportion of zero values,[4] as used in prior studies.[5, 6]

In the first part, logistic regression, incorporating propensity scores and other baseline variables as covariates in the model, was used to predict the probability of incurring any positive costs. In the second part, a generalized linear model with a gamma distribution and log link and incorporating propensity scores and other baseline variables as covariates in the model was used to estimate costs among patients with positive (non-zero) costs; combining predictions from both models generated the mean cost estimates for each of the study cohorts. Each model was fitted separately, and their predictions were combined to derive mean estimated costs.

A recycled predictions approach was then used to estimate absolute cost differences between groups with and without HZ (i.e., between the UC+/HZ+ and UC+/HZ– and between the CD+/HZ+ and CD+/HZ– cohorts), with 95% CIs estimated using non-

parametric bootstrap procedures with 499 replications. For adjusted cost differences, 95% CIs were estimated using non-parametric bootstrap procedures with 499 replications.

Table S2. Baseline demographics and clinical characteristics

	UC			CD		
	UC+/HZ+ (N = 431)	UC+/HZ– (N = 10 285)	Standardized difference[a]	CD+/HZ+ (N = 435)	CD+/HZ– (N = 9797)	Standardized difference[a]
**Demographics**						
**Year of the index date, n (%)**						
2016	10 (2.3)	197 (1.9)	2.8%	13 (3.0)	302 (3.1)	0.5%
2017	94 (21.8)	3029 (29.5)	17.5%	117 (26.9)	3390 (34.6)	16.7%
2018	275 (63.8)	6416 (62.4)	2.9%	256 (58.9)	5567 (56.8)	4.1%
2019	52 (12.1)	643 (6.3)	20.2%	49 (11.3)	538 (5.5)	20.8%
**Age at index date, years, mean ± SD**	65.3 ± 15.1	60.0 ± 17.5	32.2%	61.0 ± 17.0	56.0 ± 18.0	28.2%
18–29, n (%)	6 (1.4)	644 (6.3)	25.4%	28 (6.4)	1034 (10.6)	14.8%
30–39, n (%)	32 (7.4)	1145 (11.1)	12.8%	38 (8.7)	1190 (12.1)	11.2%
40–49, n (%)	40 (9.3)	1251 (12.2)	9.3%	49 (11.3)	1348 (13.8)	7.5%
18–49, n (%)	78 (18.1)	3040 (29.6)	26.9%	115 (26.4)	3572 (36.5)	21.6%
50–64, n (%)	104 (24.1)	2200 (21.4)	6.5%	105 (24.1)	2480 (25.3)	2.7%
≥65, n (%)	249 (57.8)	5045 (49.1)	17.5%	215 (49.4)	3745 (38.2)	22.6%
**Female, n (%)**	258 (59.9)	5602 (54.5)	10.9%	270 (62.1)	5518 (56.3)	11.7%
**Geographic region, n (%)**						
South	141 (32.7)	4275 (41.6)	18.3%	176 (40.5)	4159 (42.5)	4.0%
West	111 (25.8)	2134 (20.7)	11.8%	99 (22.8)	1733 (17.7)	12.6%
Midwest	124 (28.8)	2359 (22.9)	13.3%	110 (25.3)	2591 (26.4)	2.6%
Northeast	55 (12.8)	1501 (14.6)	5.3%	50 (11.5)	1305 (13.3)	5.5%
Unknown	0 (0.0)	16 (0.2)	5.6%	0 (0.0)	9 (0.1)	4.3%
**Insurance type, n (%)**						
Medicare Advantage	253 (58.7)	5354 (52.1)	13.4%	251 (57.7)	4729 (48.3)	18.9%
Commercial	178 (41.3)	4931 (47.9)	13.4%	184 (42.3)	5068 (51.7)	18.9%
**Clinical characteristics**						
**CCI, mean ± SD**	1.6 ± 2.2	1.1 ± 1.7	25.2%	1.4 ± 1.8	1.2 ±1.7	14.4%
0, n (%)	196 (45.5)	5592 (54.4)	17.8%	195 (44.8)	5193 (53.0)	16.4%
1, n (%)	72 (16.7)	1655 (16.1)	1.7%	81 (18.6)	1641 (16.8)	4.9%
2–4, n (%)	115 (26.7)	2463 (23.9)	6.3%	128 (29.4)	2417 (24.7)	10.7%
≥5, n (%)	48 (11.1)	575 (5.6)	20.0%	31 (7.1)	546 (5.6)	6.4%
**CCI conditions, n (%)**						
Chronic pulmonary disease	98 (22.7)	2094 (20.4)	5.8%	112 (25.7)	2128 (21.7)	9.5%
Peripheral vascular disease	84 (19.5)	1412 (13.7)	15.5%	73 (16.8)	1180 (12.0)	13.5%
Moderate or severe renal disease	77 (17.9)	1173 (11.4)	18.3%	66 (15.2)	1180 (12.0)	9.1%
Diabetes (type 1 or 2) with end-organ damage	64 (14.8)	891 (8.7)	19.2%	44 (10.1)	753 (7.7)	8.5%
Diabetes (type 1 or 2) without end-organ damage	44 (10.2)	1127 (11.0)	2.4%	41 (9.4)	934 (9.5)	0.4%
Heart failure	60 (13.9)	821 (8.0)	19.0%	40 (9.2)	744 (7.6)	5.8%
Cerebrovascular disease	50 (11.6)	874 (8.5)	10.3%	31 (7.1)	745 (7.6)	1.8%
Rheumatologic disease	43 (10.0)	583 (5.7)	16.0%	52 (12.0)	635 (6.5)	18.9%
Any malignant tumor	42 (9.7)	846 (8.2)	5.3%	43 (9.9)	732 (7.5)	8.6%
Mild liver disease	32 (7.4)	885 (8.6)	4.3%	49 (11.3)	980 (10.0)	4.1%
Myocardial infarction	31 (7.2)	428 (4.2)	13.1%	31 (7.1)	428 (4.4)	11.8%
Peptic ulcer disease	12 (2.8)	258 (2.5)	1.7%	9 (2.1)	334 (3.4)	8.2%
Dementia	15 (3.5)	286 (2.8)	4.0%	6 (1.4)	234 (2.4)	7.4%
Metastatic solid tumor	14 (3.2)	113 (1.1)	14.7%	6 (1.4)	120 (1.2)	1.4%
Hemiplegia	6 (1.4)	104 (1.0)	3.5%	2 (0.5)	72 (0.7)	3.6%
Moderate or severe liver disease	7 (1.6)	77 (0.7)	8.1%	2 (0.5)	73 (0.7)	3.7%
AIDS	1 (0.2)	38 (0.4)	2.5%	4 (0.9)	25 (0.3)	8.7%
**Comorbidities potentially associated with HZ, n (%)**	70 (16.2)	953 (9.3)	20.9%	80 (18.4)	1145 (11.7)	18.8%
Sicca syndrome [Sjögren]	5 (1.2)	66 (0.6)	5.5%	5 (1.1)	78 (0.8)	3.6%
Other interstitial lung disease	18 (4.2)	141 (1.4)	17.1%	10 (2.3)	137 (1.4)	6.7%
Systemic lupus erythematosus	9 (2.1)	92 (0.9)	9.8%	12 (2.8)	104 (1.1)	12.4%
Psoriasis	12 (2.8)	246 (2.4)	2.5%	22 (5.1)	354 (3.6)	7.1%
Psoriatic arthritis	4 (0.9)	68 (0.7)	3.0%	4 (0.9)	82 (0.8)	0.9%

Ankylosing spondylitis	4 (0.9)	78 (0.8)	1.9%	8 (1.8)	130 (1.3)	4.1%
Sarcoidosis	3 (0.7)	28 (0.3)	6.1%	2 (0.5)	28 (0.3)	2.9%
Giant cell arteritis	1 (0.2)	24 (0.2)	0.0%	2 (0.5)	14 (0.1)	5.8%
Idiopathic pulmonary fibrosis	2 (0.5)	10 (0.1)	6.9%	1 (0.2)	4 (0.0)	5.1%
Multiple sclerosis	4 (0.9)	46 (0.4)	5.8%	4 (0.9)	69 (0.7)	2.4%
Wegener's granulomatosis	0 (0.0)	6 (0.1)	3.4%	0 (0.0)	2 (0.0)	2.0%
Scleroderma	1 (0.2)	1 (0.0)	6.4%	0 (0.0)	4 (0.0)	2.9%
Rheumatoid arthritis	31 (7.2)	380 (3.7)	15.4%	38 (8.7)	441 (4.5)	17.0%
**Additional immunosuppressive conditions, n (%)**	65 (15.1)	1461 (14.2)	2.5%	102 (23.4)	2215 (22.6)	2.0%
Use of chemotherapy for solid and hematological malignancies in prior 6 months	62 (14.4)	1332 (13.0)	4.2%	94 (21.6)	2125 (21.7)	0.2%
Solid organ transplant	2 (0.5)	112 (1.1)	7.1%	4 (0.9)	77 (0.8)	1.5%
Symptomatic HIV	1 (0.2)	38 (0.4)	2.5%	4 (0.9)	25 (0.3)	8.7%
Hematopoietic stem-cell transplant	1 (0.2)	4 (0.0)	5.3%	0 (0.0)	9 (0.1)	4.3%
**Use of IBD-related medications, n (%)**	246 (57.1)	5719 (55.6)	3.0%	257 (59.1)	5705 (58.2)	1.7%
5-ASA	144 (33.4)	3981 (38.7)	11.0%	67 (15.4)	1745 (17.8)	6.5%
Systemic steroids	72 (16.7)	1284 (12.5)	12.0%	86 (19.8)	1471 (15.0)	12.5%
Anti-tumor necrosis factor biologics	55 (12.8)	1122 (10.9)	5.7%	103 (23.7)	2490 (25.4)	4.0%
Thiopurines	48 (11.1)	790 (7.7)	11.8%	75 (17.2)	1151 (11.7)	15.6%
Methotrexate	8 (1.9)	139 (1.4)	4.0%	15 (3.4)	237 (2.4)	6.1%
Ustekinumab	1 (0.2)	14 (0.1)	2.2%	15 (3.4)	245 (2.5)	5.6%
Vedolizumab	11 (2.6)	338 (3.3)	4.4%	8 (1.8)	433 (4.4)	14.8%
Janus kinase inhibitors	2 (0.5)	11 (0.1)	6.7%	1 (0.2)	5 (0.1)	4.8%
**IBD management prior to index date, n (%)**						
No therapy, 5-ASA, or budesonide	280 (65.0)	7504 (73.0)	17.3%	220 (50.6)	5336 (54.5)	7.8%
No therapy, sulfasalazine, or budesonide	208 (48.3)	5093 (49.5)	2.5%	194 (44.6)	4405 (45.0)	0.7%
**Markers of IBD severity prior to index date, n (%)**	109 (25.3)	2192 (21.3)	9.4%	109 (25.1)	2464 (25.2)	0.2%
Weight loss	27 (6.3)	538 (5.2)	4.4%	27 (6.2)	670 (6.8)	2.6%
Malnutrition	20 (4.6)	252 (2.5)	11.8%	6 (1.4)	344 (3.5)	13.8%
Anemia	90 (20.9)	1814 (17.6)	8.2%	92 (21.1)	1975 (20.2)	2.4%
**Surgeries and procedures, n (%)**	181 (42.0)	4443 (43.2)	2.4%	154 (35.4)	3639 (37.1)	3.6%
Endoscopy or colonoscopy, n (%)	178 (41.3)	4398 (42.8)	3.0%	149 (34.3)	3497 (35.7)	3.0%
Strictureplasty	0 (0.0)	0 (0.0)	0.0%	0 (0.0)	9 (0.1)	4.3%
Bowel resection	3 (0.7)	67 (0.7)	0.5%	4 (0.9)	135 (1.4)	4.3%
Colectomy	5 (1.2)	92 (0.9)	2.6%	12 (2.8)	261 (2.7)	0.6%
Surgery for intestinal or anorectal fistula	1 (0.2)	3 (0.0)	5.6%	0 (0.0)	23 (0.2)	6.9%
Surgery for abdominal or pelvic abscess	1 (0.2)	13 (0.1)	2.5%	0 (0.0)	17 (0.2)	5.9%
Proctocolectomy and restorative proctocolectomy	1 (0.2)	48 (0.5)	4.0%	1 (0.2)	15 (0.2)	1.8%

Patient demographic characteristics were identified as of the index date. Clinical characteristics were assessed during the 12-month period strictly prior to the index date.

[a] Standardized differences of 20%, 50%, and 80% suggest small, medium, and large differences between cohorts, respectively.[3]

Abbreviations: 5-ASA; aminosalicylate; AIDS, acquired immunodeficiency syndrome; CCI, modified Charlson–Quan comorbidity index; CD, Crohn's disease; HIV, human immunodeficiency virus; HZ, herpes zoster; IBD, inflammatory bowel disease; SD, standard deviation; TNF, tumor necrosis factor; UC, ulcerative colitis

UC+/HZ+ and UC+/HZ–, patients with UC with and without HZ, respectively; CD+/HZ+ and CD+/HZ–, patients with CD with and without HZ, respectively

Table S3. Baseline healthcare resource utilization and costs

	UC			CD		
	UC+/HZ+ (N = 431)	UC+/HZ– (N = 10 285)	Standardized difference[a]	CD+/HZ+ (N = 435)	CD+/HZ– (N = 9797)	Standardized difference[a]
**HCRU**						
**Any all-cause healthcare service use, n (%)**	431 (100.0)	10 280 (100.0)	3.1%	434 (99.8)	9791 (99.9)	4.4%
**All-cause medical service use, n (%)**	431 (100.0)	10 278 (99.9)	3.7%	434 (99.8)	9788 (99.9)	3.4%
Inpatient stays, n (%)	125 (29.0)	2581 (25.1)	8.8%	137 (31.5)	3101 (31.7)	0.3%
Emergency department visits, n (%)	174 (40.4)	3464 (33.7)	13.9%	197 (45.3)	3874 (39.5)	11.6%
Outpatient visits, n (%)	431 (100.0)	10 271 (99.9)	5.2%	433 (99.5)	9766 (99.7)	2.3%
Other resource use, n (%)	247 (57.3)	5076 (49.4)	15.9%	244 (56.1)	4804 (49.0)	14.1%
**Pharmacy visits, n (%)**	390 (90.5)	9305 (90.5)	0.1%	390 (89.7)	8851 (90.3)	2.3%
**Costs (US$ 2020) PPPY, mean ± SD [median]**						
**All-cause healthcare service cost**	$58 230 ± 83 680 [$25 410]	$49 526 ± 84 736 [$19 045]	10.3%	$72 849 ± 87 582 [$49 475]	$65 983 ± 87 112 [$43 602]	7.9%
Among patients with costs >$0	$58 230 ± 83 680 [$25 410]	$49 555 ± 84 746 [$19 069]	10.3%	$73 017 ± 87 613 [$49 771]	$66 030 ± 87 117 [$43 694]	8.0%
**All-cause medical service cost**	$48 295 ± 80 703 [$15 612]	$40 290 ± 81 614 [$9598]	9.9%	$54 609 ± 78 839 [$21 184]	$50 667 ± 79 403 [$20 610]	5.0%
Among patients with costs >$0	$48 295 ± 80 703 [$15 612]	$40 324 ± 81 631 [$9614]	9.8%	$54 735 ± 78 886 [$21 324]	$50 722 ± 79 418 [$20 641]	5.1%
Inpatient costs	$26 119 ± 65 580 [$0]	$22 609 ± 70 648 [$0]	5.1%	$28 487 ± 65 024 [$0]	$25 691 ± 64 398 [$0]	4.3%
Among patients with costs >$0	$90 061 ± 95 440 [$55 506]	$90 179 ± 117 451 [$53 752]	0.1%	$90 452 ± 88 581 [$65 687]	$81 234 ± 92 671 [$51 989]	10.2%
Emergency department costs	$2565 ± 5959 [$0]	$2231 ± 6834 [$0]	5.2%	$3206 ± 7391 [$0]	$3 087 ± 10 342 [$0]	1.3%
Among patients with costs >$0	$6353 ± 8003 [$4217]	$6624 ± 10 468 [$3694]	2.9%	$7079 ± 9665 [$4377]	$7807 ± 15 286 [$4025]	5.7%
Outpatient costs	$17 802 ± 31 980 [$6829]	$14 370 ± 27 161 [$5306]	11.6%	$21 631 ± 33 286 [$8166]	$20 083 ± 34 670 [$7328]	4.6%
Among patients with costs >$0	$17 802 ± 31 980 [$6829]	$14 390 ± 27 174 [$5321]	11.5%	$21 731 ± 33 330 [$8166]	$20 147 ± 34 706 [$7384]	4.7%
Other resource use costs	$1810 ± 9574 [$130]	$1080 ± 5687 [$0]	9.3%	$1285 ± 6570 [$106]	$1806 ± 8497 [$0]	6.9%
Among patients with costs >$0	$3158 ± 12 487 [$507]	$2189 ± 7944 [$400]	9.3%	$2291 ± 8648 [$506]	$3682 ± 11 847 [$475]	13.4%
**Pharmacy costs**	$9935 ± 18 608 [$3377]	$9236 ± 20 117 [$3360]	3.6%	$18 240 ± 32 863 [$3207]	$15 317 ± 35 664 [$2803]	8.5%
Among patients with costs >$0	$10 979 ± 19 268 [$4322]	$10 208 ± 20 914 [$4304]	3.8%	$20 345 ± 34 088 [$4605]	$16 954 ± 37 150 [$3729]	9.5%

HCRU and related costs were assessed during the 12-month period strictly prior to the index date. Cost data were adjusted to 2020 US$ values using the medical care component of the US Consumer Price Index.

[a] Standardized differences of 20%, 50%, and 80% suggest small, medium, and large differences between cohorts, respectively.[3]

Other resource use and related costs include durable medical equipment, home/hospice health visits, services and supplies, and transportation services.

Abbreviations: CD, Crohn's disease; HCRU, healthcare resource utilization; PPPY, per person per year; SD, standard deviation; UC: ulcerative colitis UC+/HZ+ and UC+/HZ–, patients with UC with and without HZ, respectively; CD+/HZ+ and CD+/HZ–, patients with CD with and without HZ, respectively

Table S4. All-cause healthcare resource utilization for patients with UC and CD with and without HZ

	UC				CD			
	UC+/HZ+ (N = 431)	UC+/HZ– (N = 10 285)	Adjusted IRR (95% CI)[a]	*P*-value[a]	CD+/HZ+ (N = 435)	CD+/HZ– (N = 9797)	Adjusted IRR (95% CI)[a]	*P*-value[a]
**First month**								
**All-cause HCRU**								
Any visits, n (%)	426 (98.8)	6844 (66.5)			435 (100.0)	7037 (71.8)		
Inpatient stays, n (%)	32 (7.4)	231 (2.2)			42 (9.7)	299 (3.1)		
ED visits, n (%)	60 (13.9)	494 (4.8)			76 (17.5)	604 (6.2)		
Outpatient visits, n (%)	412 (95.6)	6387 (62.1)			424 (97.5)	6612 (67.5)		
Other, n (%)	92 (21.3)	1492 (14.5)			79 (18.2)	1440 (14.7)		
**HCRU incidence rate[b]**								
Any visits	3.69	1.95	1.72 (1.56, 1.89)	**< .001**	3.90	2.19	1.76 (1.60, 1.93)	**< .001**
Inpatient stays	0.09	0.02	2.87 (1.93, 4.27)	**< .001**	0.11	0.03	3.34 (2.38, 4.70)	**< .001**
ED visits	0.17	0.08	2.66 (1.74, 4.05)	**< .001**	0.27	0.10	3.31 (2.32, 4.71)	**< .001**
Outpatient visits	3.03	1.59	1.73 (1.56, 1.91)	**< .001**	3.20	1.74	1.78 (1.62, 1.95)	**< .001**
Other	0.39	0.26	1.34 (1.02, 1.77)	**.038**	0.33	0.32	1.04 (0.76, 1.41)	.823
**First 3 months**								
**All-cause HCRU**								
Any visits, n (%)	430 (99.8)	9177 (89.2)			435 (100.0)	9020 (92.1)		
Inpatient stays, n (%)	49 (11.4)	599 (5.8)			65 (14.9)	748 (7.6)		
ED visits, n (%)	86 (20.0)	1160 (11.3)			115 (26.4)	1429 (14.6)		
Outpatient visits, n (%)	427 (99.1)	8960 (87.1)			433 (99.5)	8839 (90.2)		
Other, n (%)	156 (36.2)	2741 (26.7)			155 (35.6)	2673 (27.3)		
**HCRU incidence rate[b]**								
Any visits	8.88	5.84	1.30 (1.20, 1.42)	**< .001**	9.36	6.46	1.36 (1.26, 1.48)	**< .001**
Inpatient stays	0.14	0.07	1.66 (1.20, 2.28)	**.002**	0.19	0.09	2.09 (1.58, 2.76)	**< .001**
ED visits	0.30	0.22	1.48 (1.07, 2.05)	**.018**	0.50	0.29	2.06 (1.57, 2.71)	**< .001**
Outpatient visits	7.24	4.74	1.30 (1.19, 1.41)	**< .001**	7.61	5.15	1.36 (1.26, 1.47)	**< .001**
Other	1.20	0.82	1.25 (0.99, 1.58)	.064	1.06	0.93	1.14 (0.90, 1.45)	.276
**First 12 months**								
**All-cause HCRU**								
Any visits, n (%)	431 (100.0)	10 173 (98.9)			435 (100.0)	9720 (99.2)		
Inpatient stays, n (%)	89 (20.6)	1672 (16.3)			114 (26.2)	2024 (20.7)		
ED visits, n (%)	161 (37.4)	3159 (30.7)			190 (43.7)	3590 (36.6)		
Outpatient visits, n (%)	431 (100.0)	10 150 (98.7)			434 (99.8)	9688 (98.9)		
Other, n (%)	267 (61.9)	5190 (50.5)			258 (59.3)	4940 (50.4)		
**HCRU incidence rate, PPPY[c]**								
Any visits	31.01	23.26	1.14 (1.06, 1.23)	**< .001**	32.38	25.36	1.16 (1.09, 1.25)	**< .001**

Inpatient stays	0.41	0.25	1.34 (1.07, 1.68)	**.012**	0.46	0.36	1.27 (1.03, 1.57)	**.025**
ED visits	0.91	0.86	1.11 (0.89, 1.38)	.348	1.43	1.16	1.28 (1.05, 1.56)	**.015**
Outpatient visits	25.04	18.86	1.13 (1.06, 1.22)	**< .001**	26.26	20.20	1.17 (1.09, 1.25)	**< .001**
Other	4.66	3.29	1.17 (0.97, 1.41)	.101	4.23	3.65	1.06 (0.87, 1.28)	.575

[a] Adjusted incidence rate ratios (aIRRs) and 95% confidence intervals (CIs) were estimated using generalized linear models, assuming a negative binomial distribution and log link, adjusting for potential confounders using the patients' propensity scores to account for potential baseline differences between cohorts. *P*-values were calculated using the negative binomial distribution.

[b] Incidence rates were calculated as the average number of HCRU events per patient.

[c] Incidence rates were calculated by dividing the number of encounters over the observation period by the patient-time observed; the incidence rates were then reported on a PPPY basis.

Other resource use or ancillary care visits include home/hospice health visits, skilled nursing facilities, transportation services, and durable medical equipment.

Abbreviations: aIRR, adjusted incidence rate ratio; CD, Crohn's disease; CI, confidence interval; ED, emergency department; HCRU, healthcare resource utilization; HZ, herpes zoster; PPPY, per person per year; UC: ulcerative colitis

UC+/HZ+ and UC+/HZ–, patients with UC with and without HZ, respectively; CD+/HZ+ and CD+/HZ–, patients with CD with and without HZ, respectively

Table S5. All-cause healthcare costs in patients with UC and CD with and without HZ

	UC			CD		
	UC+/HZ+ (N = 431)	UC+/HZ– (N = 10 285)	Adjusted cost difference (95% CI)[a,b]	CD+/HZ+ (N = 435)	CD+/HZ– (N = 9797)	Adjusted cost difference (95% CI)[a,b]
**First month ($US 2020) mean ± SD**						
Total all-cause healthcare service costs	$6515 ± 16 899	$3679 ± 13 713	$2189 (886, 3975)	$9910 ± 35 428	$5195 ± 14 287	$3774 (1829, 6972)
All-cause medical service costs	$5547 ± 16 608	$2890 ± 13 574	$1854 (711, 3608)	$8506 ± 35 292	$3763 ± 13 498	$3236 (1594, 5683)
Inpatient costs	$3278 ± 15 636	$1335 ± 12 468	$1284 (307, 2683)	$6162 ± 34 811	$1563 ± 12 147	$2873 (1504, 5443)
ED costs	$376 ± 1431	$166 ± 1214	$205 (106, 358)	$489 ± 1636	$231 ± 1438	$297 (161, 472)
Outpatient costs	$1673 ± 3649	$1284 ± 4588	$346 (69, 716)	$1740 ± 4133	$1810 ± 5137	$130 (—215, 523)
Other resource use costs	$220 ± 2195	$105 ± 869	$34 (—17, 89)	$115 ± 777	$158 ± 1102	—$22 (—75, 58)
Pharmacy costs	$968 ± 2596	$789 ± 2103	$78 (—57, 233)	$1405 ± 3832	$1433 ± 4876	$79 (—257, 451)
**First 3 months ($US 2020) mean ± SD**						
Total all-cause healthcare service costs	$14 552 ± 27 096	$10 721 ± 26 041	$2613 (424, 5057)	$22 571 ± 52 049	$15 505 ± 30 150	$5556 (2396, 11 600)
All-cause medical service costs	$11 826 ± 25 912	$8305 ± 25 399	$2194 (89, 4523)	$17 894 ± 51 322	$11 205 ± 28 407	$5011 (2189, 9387)
Inpatient costs	$5966 ± 22 308	$3767 ± 21 928	$1480 (—157, 3319)	$10 537 ± 47 605	$4682 ± 24 178	$4059 (1511, 9512)
ED costs	$669 ± 2081	$490 ± 2573	$153 (0, 399)	$1081 ± 3608	$698 ± 2865	$298 (62, 681)
Outpatient costs	$4700 ± 9627	$3718 ± 9889	$537 (—163, 1355)	$5980 ± 10 682	$5325 ± 11 677	$908 (—151, 1996)
Other resource use costs	$492 ± 3385	$331 ± 1984	$43 (—53, 201)	$296 ± 1347	$501 ± 2694	—$40 (—152, 79)
Pharmacy costs	$2726 ± 7359	$2415 ± 5782	$33 (—386, 429)	$4676 ± 9493	$4300 ± 10 732	$748 (—221, 1822)
**First 12 months ($US2020) PPPY, mean ± SD**						
Total all-cause healthcare service costs	$57 538 ± 101 307	$43 162 ± 73 558	$8486 (—176, 17 399)	$71 413 ± 96 127	$61 949 ± 90 701	$5583 (—2754, 15 503)
All-cause medical service costs	$46 817 ± 97 163	$33 514 ± 70 572	$7864 (—880, 17 703)	$52 149 ± 86 489	$43 973 ± 79 868	$5569 (—1300, 13 093)
Inpatient costs	$24 629 ± 85 106	$15 217 ± 55 818	$5228 (—1221, 12 639)	$25 327 ± 71 800	$18 360 ± 60 807	$5826 (262, 12 048)
ED costs	$2400 ± 6490	$1931 ± 6384	$328 (—161, 842)	$3126 ± 9205	$2909 ± 10 150	$96 (—424, 913)
Outpatient costs	$17 855 ± 31 699	$15 020 ± 31 800	$1116 (—1081, 3436)	$22 382 ± 33 940	$20 667 ± 37 561	$1626 (—1231, 4808)
Other resource use costs	$1932 ± 12 382	$1346 ± 7056	$138 (—217, 851)	$1315 ± 5796	$2038 ± 9498	—$232 (—605, 199)
Pharmacy costs	$10 721 ± 30 230	$9648 ± 20 435	—$409 (—1755, 1054)	$19 263 ± 37 224	$17 976 ± 42 810	$740 (—2669, 4316)

[a] Healthcare costs for each of the study cohorts were estimated using a two-part model. (1) In the first part, the probability of observing a positive cost was modeled using logistic regression; (2) in the second part, a generalized linear model with a gamma distribution and log link was used to predict costs among patients with positive costs. Both models accounted for the patients' propensity scores of being in the UC+/HZ+ or CD+/HZ+ cohorts and relevant baseline characteristics. Mean adjusted cost differences between the cohorts with and without HZ were then estimated using a recycled predictions approach.
[b] 95% CIs for adjusted cost difference comparisons were estimated from non-parametric bootstrap procedures with 499 replications.
Other resource use costs include home health/hospice visits, skilled nursing facilities, and use of transportation services and durable medical equipment.
Cost data were adjusted to 2020 US$ values using the medical care component of the US Consumer Price Index.

Abbreviations: CD, Crohn’s disease; CI, confidence interval; ED, emergency department; HCRU, healthcare resource utilization; HZ, herpes zoster; PPPY, per person per year; SD, standard deviation; UC, ulcerative colitis

UC+/HZ+ and UC+/HZ–, patients with UC with and without HZ, respectively; CD+/HZ+ and CD+/HZ–, patients with CD with and without HZ, respectively

Table S6. Codes used to identify medication use in study cohorts

Code Type	Code	Description	Code Type	Code	Description
**Pharmacy claims and utilization**					
**Aminosalicylic acid (5-ASA)**			**Methotrexate**		
GPI	09 00 00 10 00	Aminosalicylic acid	GPI	21 30 00 50 00	Methotrexate
GPI	52 50 00 30	Mesalamine	GPI	66 25 00 50 00	Methotrexate (Antirheumatic)
GPI	52 50 00 40 10	Olsalazine	GPI	21 30 00 50 10	Methotrexate sodium
GPI	52 50 00 20 10	Balsalazide	GPI	66 25 00 50 10	Methotrexate sodium (Antirheumatic)
GPI	52 50 00 60 00	Sulfasalazine	HCPCS	J8610	Methotrexate oral 2.5 mg
			HCPCS	J9250	Methotrexate sodium, 5 mg
			HCPCS	J9260	Methotrexate sodium, 50 mg
**Thiopurines**			**Biologics**		
GPI	21 30 00 40 00	Mercaptopurine	GPI	52 50 50 40	Infliximab
GPI	96 66 50 53 89	Mercaptopurine (bulk)	GPI	66 27 00 15	Adalimumab
GPI	96 66 50 53 90	Mercaptopurine monohydrate	GPI	66 27 00 40 00	Golimumab
GPI	99 40 60 10 00	Azathioprine	GPI	52 50 50 20 10	Certolizumab
GPI	99 40 60 10 10	Azathioprine sodium	GPI	52 50 30 80 00	Vedolizumab
GPI	21 30 00 60 00	Thioguanine	GPI	52 50 40 70 00	Ustekinumab (IV)
GPI	96 80 56 27 30	Thioguanine (bulk)	GPI	90 25 05 85 00	Ustekinumab
HCPCS	S0108	Mercaptopurine, oral, 50 mg	HCPCS	J1745	Injection, infliximab, excludes biosimilar, 10 mg
HCPCS	J7500	Azathioprine, oral, 50 mg	HCPCS	Q5102	Injection, Infliximab, Biosimilar, 10 mg
HCPCS	J7501	Azathioprine, parenteral, 100 mg	HCPCS	Q5103	Injection, infliximab-dyyb, biosimilar, (inflectra), 10 mg
**Janus kinase Inhibitor**			HCPCS	Q5104	Injection, infliximab-abda, biosimilar, (renflexis), 10 mg
GPI	66 60 30 65 10	Tofacitinib citrate	HCPCS	Q5109	Injection, infliximab-qbtx, biosimilar, (ixifi), 10 mg
GPI	96 80 70 20 10	Tofacitinib citrate (bulk)	HCPCS	Q5121	Injection, infliximab-axxq, biosimilar, (avsola), 10 mg
			HCPCS	J0135	Injection, adalimumab, 20 mg
			HCPCS	J1602	Injection, golimumab, 1 mg, for intravenous use
			HCPCS	J0717	Injection, certolizumab pegol, 1 mg
			HCPCS	J3380	Injection, vedolizumab, 1 mg
			HCPCS	C9026	Injection, vedolizumab, 1 mg (Deleted 2016-01-01)
			HCPCS	J3358	Ustekinumab, for intravenous injection, 1 mg

			HCPCS	J3357	Ustekinumab, for subcutaneous injection, 1 mg
**Systemic steroids**			**Herpes zoster vaccination**		
GPI	22 10 00 30	Methylprednisolone	CPT	90736	Zoster (shingles) vaccine (HZV), live, for subcutaneous injection
GPI	22 10 00 40	Prednisolone	CPT	90750	Zoster (shingles) vaccine (HZV), recombinant, subunit, adjuvanted, for intramuscular use
GPI	22 10 00 45	Prednisone	HCPCS	G2160	Patient received at least one dose of the herpes zoster live vaccine or two doses of the herpes zoster recombinant vaccine (at least 28 days apart) anytime on or after the patient's 50th birthday before or during the measurement period
GPI	22 10 00 25	Hydrocortisone	HCPCS	G2161	Patient had prior adverse reaction caused by zoster vaccine or its components any time during or before the measurement period
GPI	96 56 88 11 30	Hydrocortisone hemisuccinate (bulk)	HCPCS	M1064	Recombinant zoster vaccine documented as administered or previously received
GPI	22 10 00 12 00	Budesonide	GPI	17 10 00 95 10	Zoster Vaccine Live
GPI	96 44 82 12 00	Budesonide (bulk)	GPI	17 10 00 95 40	Zoster Vaccine Recombinant Adjuvanted
GPI	89 15 00 07 00	Budesonide (intrarectal)	NDC	50090-3372-00	*Shingrix*, 1 KIT IN 1 KIT (50090-3372-0) * 0.5 mL IN 1 VIAL (58160-828-03) * 0.5 mL IN 1 VIAL (58160-829-03)
HCPCS	J1020	Injection, methylprednisolone acetate, 20 mg	NDC	50090-5147-00	*Shingrix*, 1 KIT IN 1 KIT (50090-5147-0) * 0.5 mL IN 1 VIAL (58160-828-01) * 0.5 mL IN 1 VIAL (58160-829-01)
HCPCS	J1030	Injection, methylprednisolone acetate, 40 mg	NDC	58160-828-01	*Shingrix*, 0.5 mL in 1 Lyophilized Antigen Vial
HCPCS	J1040	Injection, methylprednisolone acetate, 80 mg	NDC	58160-829-01	*Shingrix*, 0.5 mL in 1 Adjuvant Suspension Vial
HCPCS	J2920	Injection, methylprednisolone sodium succinate, up to 40 mg	NDC	58160-819-12	*Shingrix*, 1 KIT in 1 CARTON * 0.5 mL in 1 VIAL (58160-828-01) *0.5 mL in 1 VIAL (58160-829-01)
HCPCS	J2930	Injection, methylprednisolone sodium succinate, up to 125 mg	NDC	58160-828-03	*Shingrix*, 0.5 mL in 1 Lyophilized Antigen Vial
HCPCS	J7509	Methylprednisolone oral, per 4 mg	NDC	58160-829-03	*Shingrix*, 0.5 mL in 1 Adjuvant Suspension Vial

HCPCS	J2650	Injection, prednisolone acetate, up to 1 ml	NDC	58160-823-11	*Shingrix*, 1 KIT in 1 CARTON * 0.5 mL in 1 VIAL (58160-828-03) * 0.5 mL in 1 VIAL (58160-829-03)
HCPCS	J7510	Prednisolone oral, per 5 mg	NDC	00006-4963-00	*Zostavax*, 1 VIAL, SINGLE-DOSE in 1 CARTON > 0.65 mL in 1 VIAL, SINGLE-DOSE
HCPCS	J7506	Prednisone, oral, per 5 mg	NDC	00006-4963-01	*Zostavax*
HCPCS	J7512	Prednisone, immediate release or delayed release, oral, 1 mg	NDC	00006-4963-41	*Zostavax*, 10 VIAL, SINGLE-DOSE in 1 CARTON > 0.65 mL in 1 VIAL, SINGLE-DOSE
HCPCS	J1700	Injection, hydrocortisone acetate, up to 25 mg			
HCPCS	J1710	Injection, hydrocortisone sodium phosphate, up to 50 mg			
HCPCS	J1720	Injection, hydrocortisone sodium succinate, up to 100 mg			
HCPCS	J2650	Injection, prednisolone acetate, up to 1 ml			

CPT, Current Procedural Terminology; HCPCS, Healthcare Common Procedure Coding System; GPI, Generic Product Identifier; NDC, National Drug Code; ICD-10-CM: International Classification of Diseases, 10th Revision, Clinical Modification; ICD-10-PCS: International Classification of Diseases, 10th Revision, Procedure Classification System

Trademarks:
Shingrix is a trademark owned by or licensed to GSK.
Zostavax is a trademark of Merck & Co., Inc.

References for Supplementary Material:

1. Manceur AM, Ding Z, Muser E, et al. Burden of Crohn's disease in the United States: Long-term healthcare and work-loss related costs. *J Med Econ*. 2020;23(10):1092–1101.
2. Shaw SY, Blanchard JF, Bernstein CN. Association between the use of antibiotics and new diagnoses of Crohn's disease and ulcerative colitis. *Am J Gastroenterol*. 2011;106(12):2133–2142.
3. Cohen J. Statistical power analysis for the behavioral sciences. 3rd ed. Mahwah, NJ: Lawrence Erlbaum Associates; 1988.
4. Deb P, Norton EC. Modeling health care expenditures and use. *Annu Rev Public Health*. 2018;39:489–505.
5. Ciminata G, Geue C, Langhorne P, et al. A two-part model to estimate inpatient, outpatient, prescribing and care home costs associated with atrial fibrillation in Scotland. *BMJ Open*. 2020;10(3):e028575.
6. Ghaswalla P, Thompson-Leduc P, Cheng WY, et al. Increased health care resource utilization and costs associated with herpes zoster among patients aged ≥50 years with chronic obstructive pulmonary disease in the United States. *Chronic Obstr Pulm Dis*. 2021;8(4):502–516.
